# Kinetics of dehydration and appreciation of the physicochemical properties of osmo‐dehydrated plum

**DOI:** 10.1002/fsn3.2191

**Published:** 2021-02-18

**Authors:** Shahnaj Pervin, Md. Gulzarul Aziz, Md. Miaruddin

**Affiliations:** ^1^ Department of Food Technology and Rural Industries Bangladesh Agricultural University Mymensingh Bangladesh; ^2^ Postharvest Technology Division Bangladesh Agricultural Research Institute Gazipur Bangladesh

**Keywords:** color, drying rate, osmo‐dehydrated plum, overall acceptability, phenolic content, texture, water activity

## Abstract

The experiment was conducted to evaluate the dehydration kinetics and quantify its effect on the various physicochemical properties of the osmo‐dehydrated plum during storage at an ambient condition. The six treatments with a combination of three different sucrose–sodium chloride concentrations and two peeling conditions were selected in the experiment. Among the treatments, peeled plum dipped into 5% NaCl solution exhibited a faster drying rate. Concerning the rehydration properties of the osmo‐dehydrated plum, the whole plum immersed into 50^0^B sucrose solution showed the highest reconstitution behavior and the lowest moisture content (wb). The highest values of water activity of 0.514 and the lowest values of texture 1.79 N‐mm^2^ were investigated in 50^0^B sucrose treated whole plum. The peeled plum obtained the highest lightness (L), redness (a*), and yellowness (b*) compared to the unpeeled plum. Osmo‐dehydrated plum with high sugar solution contained more sugar and less total phenolic content nevertheless using only 5% NaCl resulted in less sugar and more total phenolic content after the treatment. The osmo‐dehydrated whole plums prepared in 50^0^B sucrose scored the highest overall acceptability (8.0, e.g., like very much) followed by the 50^0^B sucrose with peeled plum envisaged the sensory evaluation analysis. In conclusion, the osmo‐dehydrated plum treated in 50^0^B sucrose and unpeeled condition performed better with a view to the overall plum quality, color, and acceptability judged by the expert panelists even after 12 months of storage at room temperature.

## Graphical abstract

Peeled plum exhibited faster drying rate along with highest lightness (L), redness (a*), and yellowness (b*) compared to the unpeeled plum. The maximum values of water activity and the lowest texture were investigated in 50^0^B sucrose treated whole plum. Osmo‐dehydrated plums prepared from the whole and peeled plum using only 5% NaCl solution resulted in less sugar and more total phenolic content. With a view to the dehydrated plum overall quality, color, and acceptability, unpeeled plum treated in 50^0^B sucrose performed better.

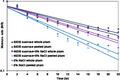

## 1 INTRODUCTION

In Bangladesh, the demand of plum (*Prunus domestica*) usually meets up by importing from other countries like India, China, and Thailand (Mozumder et al., 2017). Spices Research Center of Bangladesh Agricultural Research Institute (BARI) released a plum variety namely “BARI Alu bukhara‐1” which is high yielding and profit potential (Anonymous, 2014), but there is no available processing method to utilization of recently produced plum in Bangladesh. Hence, the suitable plum processing technique is needed. Various food processing techniques can be engaged to preserve fruits and vegetables; and dehydration is one of the most important operations that are widely practiced because of long time consumption (Chavan & Amarowicz, 2012). In recent years, there is growing demands by the customer for osmo‐dehydrated plum with a comparatively long‐life span, which preserve the attributes of fresh plum. In the case of fruit like plum, to obtain a fresh like plum implies certain operations such as whole or peeled and dip in sucrose–sodium chloride solution or often, partial dehydration of the plum. Osmotic dehydration has been the main effective method of dehydration with some advantages over other methods of drying. Therefore, osmotic dehydration has received remarkable attention in the use of moderate operating temperature, low energy process, reduced loss of volatile compounds, and better quality of the developed dehydrated plum (Lama, 2018).

Osmotic dehydration is a preservation process that is sometimes used as a pretreatment to enhance the quality of conventional dried plum (Monnerat et al., 2006). One of the most exoteric osmotic agents for fruits is sucrose because of its low cost, but other agents, such as glucose or concentrated fruit juices, are also used (Mandala et al., 2005; Rastogi et al., 2002). Osmotic dehydration is a counter flow process that results in solids gain, improving the textural and rheological properties of plum and other related fruits. It elevated the overall quality of plums as compared to conventional drying methods (Birwal et al., 2016). Consequently, the characteristics of the osmo‐dehydrated plum can be varied by controlling temperature, sugar syrup concentration, the concentration of osmosis solution, time of osmosis, etc., which require osmotic concentration process faster. For fruits, the most commonly used osmotic agents were sucrose, glucose, and NaCl for vegetables (Chavan & Amarowicz, 2012). Bongirwar and Sreenivasan (1977) pointed out that the high temperature above 60°C modifies the tissue characteristics favoring impregnation phenomena and thus solid gain. Rahman and Lamb (1991) indicated the rate of sucrose diffusion is a function of solute concentration and temperature. As osmotic dewatering is a simultaneous counter‐current mass transfer process, there are many changes in the chemical composition of food after osmotic treatment (Lewicki and Porzecka‐Pawlak, 2005; Sablani and Rahman, 2003; Robert, 2008).

The process of reintroducing water to dried foods to reach similar water levels as in their initial state is called rehydration (Vega et al., 2009). The factor which affects rehydration of any osmo‐dehydrated plum is the chemical composition of the dried fruits and vegetables, method and conditions of dehydration, solvent medium, and temperature (Taiwo & Adeyemi, 2009). In view of the physicochemical properties of fresh plum that could assist the dehydration and rehydrating properties of the osmo‐dehydrated plum, this might be established in the present research.

The kinetics of dehydration, rehydration properties, and quality characteristics of dehydrated fruits such as mango, guava and reola (Kumar & Sagar, 2014), banana, apple, apple slices (Ghasemkhani et al., 2016), kiwifruit (Maskan, 2001), and longan (Chunthaworn et al., 2012). From the viewpoints of the above studies, the research on dehydration behavior of plum and physicochemical quality attributes of osmo‐dehydrated plum is scare. Therefore, the effect of processing variables on the dehydration kinetics of plum along with the assessment of the physicochemical and rehydration properties of the osmo‐dehydrated plum produced from fresh plum is the objectives set for the study.

## 2 MATERIALS AND METHODS

### 2.1 Collection and method of processing of plum

The plum fruits were collected from the Spices Research Centre, Bangladesh Agricultural Research Institute, Gazipur. The fruits were sorted, washed, and cleaned. Then, it was blanched in boiling water for 5 min and the plum was peeled by hand. The whole and peeled plum were dipped into 50^0^B sucrose, 45^0^B sucrose plus 5% sodium chloride solution, and only 5% sodium chloride solution for 1.5 hr. Then, they were heated at 100°C for 2 min. For the preservation purpose, the KMS (1 g/L) and acetic acid (6 g/L) were added. The dehydration temperature was maintained at 60°C. After drying, the fruits were preserved in glass containers. Finally, the dehydrated fruits were analyzed at an interval of 3 months during storage for 1 year at room temperature.

There were six treatments in the experiment such as T_1_ = 50^0^B sucrose in whole plum; T_2_ = 50^0^B sucrose in peeled plum; T_3_ = 45^0^B sucrose + 5% NaCl in whole plum; T_4_ = 45^0^B sucrose + 5% NaCl in peeled plum; T_5_ = 5% NaCl in whole plum; and T_6_ = 5% NaCl in peeled plum.

### 2.2 Mechanical drying

Cabinet dryer, Model OV‐165 (Gallen Kamp Company) was used for the dehydration of the plum. The dryer consists of a chamber in which wetted plum could be placed. Air was blown by a fan pass through a heater and then across the trays of plums to be dried. The velocity of air was recorded (0.6 m/s) by an Anemometer. The dehydrated plum was taken for the determination of moisture content. Fresh plums (without peel and peel) at a constant loading density (0.5 kg/ft^2^) were placed in trays in the drier, and drying was commenced in the drier at a constant air velocity (0.6 m/s) and a specific air‐dry bulb temperature of 60°C. Weight loss was used as a measure of the extent of drying.

Fick's second law of diffusion (for plum dehydration) is applied for describing mass transfer during drying. The expression is as follows:

$$\frac{\delta M}{\delta t}=\Delta^{2}D_{e}M$$

where, *M* = Moisture content (dry basis); *t* = Time; *D*_e_ = Effective diffusion coefficient.

The solution for an infinite slab, when dried from one major face (Booker et al., 1974; Crank, 1975; Islam, 1980) is:

(1) $$\text{MR}=\frac{M_{t}‐M_{0}}{M_{0}‐M_{t}}\;=\;\frac{8}{\pi^{2}}\sum_{n\;=0}^{\alpha }\exp.\left[\frac{‐{\left(2n+1\right)}^{2}\pi^{2}D_{e}t}{L^{2}}\right]$$

For low *M_e_* values and for moisture ratio, MR < 0.6 Equation (1) reduces to:

(2) $$\frac{M_{t}}{M_{0}}=\frac{8}{\pi^{2}}e\frac{‐\pi^{2}D_{e}t}{L^{2}}=\frac{8}{\pi^{2}}e^{‐\text{me}}$$

where, $m=\frac{\pi^{2}D_{e}}{L^{2}}=\text{drying}\;\text{rate}\;\text{constant,}\;{\text{sec}}^{‐1}$

Rearranging equation (2) gives:

(3) $$\text{In}\frac{M_{t}}{M_{0}}=\text{In}\frac{8}{\pi^{2}}‐\text{mt}$$

Consequently, a straight line was obtained when plotting in MR versus time (*t*).

### 2.3 Rehydration properties

#### 2.3.1 Determination of dehydration ratio

The dehydration ratio of the dried plum (without peel and peel) was calculated by the following formula:

$$\text{Dehydration}\;\text{ratio}=\frac{\text{Weight of prepared}\;\text{material}\;\text{before}\;\text{drying}}{\text{Weight of dried plums}}$$

#### 2.3.2 General procedure for rehydration (reconstitution)

Rehydration means refreshing the dehydrated or dried plums in water. Six beakers of each 500 ml capacity were taken, and 100 ml of hot water (60°C) and 5 g of the dried samples were poured into each beaker. The wetted plum weight was taken in 5 min intervals up to 30 min. During the weighing process, the liquid portion was drained off and solid contents were transferred to a 4‐inch diameter Buchner funnel separately fitted with filter paper to remove excess water from the plum by applying a gentle suction for a few seconds. The rehydrated materials were removed from the funnel, and the weight is taken individually, and finally, the following relations were found:

$$\text{Rehydration}\;\text{ratio}=\frac{\text{Weight of rehydrated}\;\text{material}}{\text{Weight}\;\text{of}\;\text{dehydrated}\;\text{material}}$$

$$\text{Co}‐\text{efficient}\;\text{of}\;\text{reconstitution}=\frac{\text{Rehydration}\;\text{ratio}}{\text{Dehydration}\;\text{ratio}}$$

### 2.4 Water activity

Water activity of the dehydrated plum was determined by the chilled mirror technique using a Novasina water activity meter (Decagon devices Inc.).

### 2.5 Measurement of osmo‐dehydrated plum color

Dehydrated plum color was determined using a tristimulus colorimeter (CR‐400, Minolta Corp., Japan) with 8‐mm aperture and C light source at two equidistant points on the equator of each sample by using CIE color system on the *L*, a*, and b* color space where *L*, a*, and b* coordinates were recorded using D65 illuminants. A 10° standard observer was used as a reference system. *L* (lightness), a* (‐greenness to + redness), and b* (‐blueness to + yellowness) are the chromaticity coordinates.

### 2.6 Measurement of texture

Osmo‐dehydrated plum texture was analyzed using cross‐sectional prove of Texture Analyzer TA.XT plus by back extrusion method. The test mode compression was used to determine the working capacity of the instrument with a test speed of 1 mm/s and distance was 2.50 cm. The data analysis was performed by Texture Exponent Lite version 6.1.14.0 software (Stable Micro System) to find out the rupture force, and it was expressed as N.

### 2.7 Measurement of sugar

Total sugar and reducing sugar were determined by Nelson (1944).

Reducing sugars were estimated as percent and calculated it as given below:

$$\text{Reducing}\;\text{sugar}\left(\%\right)=\frac{\text{Factor}\times \text{Dilution}}{\text{Titre}\;\text{value}\times \text{Weight}\;\text{of}\;\text{sample}}\times 100.$$

The total sugar was estimated as percent and calculated as given under:

$$\text{Total}\;\text{invert}\;\text{sugar}\left(\%\right)=\frac{\text{Factor}\times \text{Dilution}}{\text{Titre}\;\times \text{Weight}\;\text{of}\;\text{sample}\;\text{taken}}\times 100.$$

$$\%\text{Sucrose}=\left(\%\text{Total invert sugars}‐\%\text{Reducing sugars}\right)\times 0.95$$

%Total sugars=(%Reducing sugars+%Sucrose)

### 2.8 Total phenol

Total phenolic content was extracted with 80% ethanol and was estimated based on their reaction with an oxidizing agent phosphomolybdate in Folin–Ciocalteau reagent under alkaline conditions (Bray & Thorpe, 1954). The developed blue color was measured at 650 nm in a UV‐VS spectrophotometer (Shimadzu, Japan). The standard curve was prepared using different concentrations (8–32 μg/ml) of catechol, and the result was expressed as mg per 100 g on a fresh weight basis.

### 2.9 Sensory evaluation

The sensory evaluation of the osmo‐dehydrated plum was carried out at every 3 months interval during storage using a sensory taste questionnaire judged by expert sensory panelists. Each treatment was assigned a letter code to avoid biases among the panelists. The samples were presented to panelists in different orders to avoid order preference among the panelists. The osmo‐dehydrated plum was rated by 10 experienced panelists who were asked to score samples based on the plum external color, off‐flavor, firmness, sweet–sour balance, and overall acceptance using a 9‐point hedonic scale.

### 2.10 Data analysis

The experiment was carried out completely randomized design (CRD), and all six treatments were replicated three times. The data were analyzed for ANOVA using computerized statistical software of R to compare the means and the level of significance of data.

## 3 RESULTS AND DISCUSSION

### 3.1 Dehydration kinetics

#### 3.1.1 Effects of peeling and sucrose–sodium chloride concentrations on dehydration time

**FIGURE 1:**
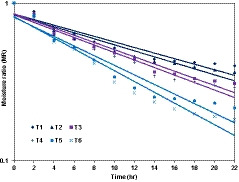
Effect of peeling and various sucrose–sodium chloride concentrations on dehydration rate of plum at a constant temperature of 60°C. T1, 50^0^B sucrose in whole plum; T2, 50^0^B sucrose in peeled plum; T3, 45^0^B sucrose + 5% NaCl in whole plum; T4, 45^0^B sucrose + 5% NaCl in peeled plum; T5, 5% NaCl in whole plum; T6, 5% NaCl in peeled plum

The fresh mature plum (whole and peeled) osmosed in different solutions was dried in the cabinet dryer at a constant temperature of 60°C using a single layer of material. The experimental data were analyzed by using Equation 3; and moisture ratio (MR) versus drying time (hr) were plotted on a semi‐log coordinate, and regression lines were drawn in Figure 1. At constant loading density and constant temperature, the faster drying was observed for peeled plum than that of the whole plum. It was noted that the plum peel has a profound influence on dehydration rate, and it offers higher resistance in both heat and mass transfer with resultant higher drying time for peel less plum. For osmo‐dehydrated plum, the drying rate constant and R‐squared values were less in 50^0^B sucrose with whole plum and more in 50^0^B sucrose with peeled plum; the same trend was observed in another treated sample for whole plum and peeled plum, respectively, as shown in Table 1. It could be concluded that the rate constant of osmo‐dehydrated peeled plum was decreased in all cases. This implies that at a specific moisture ratio, more amount of water is evaporated per unit area for a given time from the samples of peeled plum than that of the whole plum. This behavior is attributed due to broader mass transfer resistance given by the plum peel compared to the rest of the plum material (i.e., starchy endosperm, tube cell, epidermis, etc.). A similar result was reported by Pervin et al. (2007) for the effect of drying on bean seeds. It was observed that the NaCl concentration in plum gave a faster drying rate than that of the sucrose concentration.

**TABLE 1:** Effect of peeling and solute concentrations on dehydration rate constant and R^2^ of dehydrated plum

Treatments	Dehydration rate constant	*R*‐squared
T_1_	0.041	0.8223
T_2_	0.044	0.8389
T_3_	0.053	0.8528
T_4_	0.055	0.8573
T_5_	0.070	0.8063
T_6_	0.079	0.8229

Abbreviations: T_1_, 50^0^B sucrose in whole plum; T_2_, 50^0^B sucrose in peeled plum; T_3_, 45^0^B sucrose + 5% NaCl in whole plum; T_4_, 45^0^B sucrose + 5% NaCl in peeled plum; T_5_, 5% NaCl in whole plum; T_6_, 5% NaCl in peeled plum.

#### 3.1.2 Rehydration characteristics of dehydrated plum

For dehydrated plum, the rehydration ratio for the peeled plum was higher than that of the whole plum for all the treated samples. For peeled plum, the highest rehydration ratio was 1.61 (T_6_) followed by the whole plum it was 1.47 (T_5_) and the same result was investigated in other treated samples. It was obvious that the plum peel has a significant effect on the rehydration of the plum. The peeled plum resulted in higher rate of drying that might have increased the rehydration rate of the plum as because of the cellular and structural disruption during drying. The reduced rate of shrinkage of the peeled plum has also influenced the attained of a higher rate of rehydration. The coefficient of reconstitution for whole and peeled plum; the highest values were 0.55 and 0.52 in the 50^0^B sucrose concentration, respectively, which was followed by the values of 0.44 and 0.43 in 45^0^B sucrose + 5% NaCl concentration, respectively, and the lowest values of 0.32 and 0.29 in only 5% NaCl concentration, respectively (Table 2), which indicated that the osmo‐dehydrated plum possessed better reconstitution properties using different sucrose concentration than that of NaCl counterparts. This behavior may be attributed to the change in the rate of drying during osmotic treatments using various solutions (Kueneman et al., 1975).

**TABLE 2:** Effect of peeling and various solutes concentrations on the rehydration characteristics of dehydrated plum

Treatments	Weight (g) of the rehydrated sample at different duration in min							Rehydration ratio for 30 min	Dehydration ratio	coefficient of reconstitution	% m.c. (wb) of rehydrated plum
	0	5	10	15	20	25	30				
T_1_	5	5.85	6.05	6.29	6.65	6.77	6.85	1.37	2.50	0.55	37.97
T_2_	5	6.26	6.45	6.77	6.88	7.23	7.15	1.43	2.74	0.52	40.04
T_3_	5	5.95	6.19	6.45	6.62	6.75	6.95	1.39	3.16	0.44	37.97
T_4_	5	6.34	6.48	6.84	6.91	7.1	7.24	1.45	3.33	0.43	40.21
T_5_	5	6.25	6.65	6.75	6.78	7.21	7.37	1.47	4.55	0.32	40.35
T_6_	5	6.31	7.2	7.51	7.71	7.65	8.07	1.61	5.56	0.29	44.89

Abbreviations: T_1_, 50^0^B sucrose in whole plum; T_2_, 50^0^B sucrose in peeled plum; T_3_, 45^0^B sucrose + 5% NaCl in whole plum; T_4_, 45^0^B sucrose + 5% NaCl in peeled plum; T_5_, 5% NaCl in whole plum; T_6_, 5% NaCl in peeled plum.

### 3.2 Physico‐chemical properties of osmo‐dehydrated plum

The osmo‐dehydrated plum was stored in an ambient condition for one year. The changes in water activity (*a_w_*) of stored osmo‐dehydrated plum was seen in Table 3. There were significant differences observed due to variation in the solute concentrations as well as the peeling condition of the plum. In case of the peeling effect, initial *a_w_* (0.50) was found the highest in the whole plum and the lowest was 0.49 in the peeled plum. During the prolonged storage, *a_w_* was increased by 20.0% and 10.2 percent in whole and peeled plum, respectively. For the effect of solute concentrations, the plum in 50^0^B sucrose showed the highest *a_w_* (0.51) followed by the plum in 45^0^B sucrose + 5% NaCl which scored the second‐highest *a_w_* (0.49). Concerning the interaction between peeling conditions and solute concentrations, the *a_w_* for the whole plum was 0.514, 0.511, and 0.479 for the treatments of T_1_, T_3,_ and T_5_, respectively, and the percent increase was 21.79%, 21.14%, and 13.78% for the same treatments, respectively, which assumed due to the presence or absence of sucrose and NaCl in the plum. It might be happened due to temperature and humidity changes round the year during storage. The highest values of *a_w_* mean the increasing rate of water content for the treated sample of 50^0^B sucrose in the whole plum. In dehydrated plum, the higher water content may decrease the browning rate by diluting the reactive components of the plum and a similar investigation was observed by Labuza and Saltmarch (1981).

**TABLE 3:** Effect of peeling and various sucrose–sodium chloride concentrations on the water activity (*a_w_*) of osmo‐dehydrated plum during storage

Factors/Treatments	*a_w_* of osmo‐dehydrated plum at different storage (months)				
	0	3	6	9	12
Peeling conditions					
Whole plum	0.50a	0.52a	0.54a	0.57a	0.60a
Peeled plum	0.49b	0.50b	0.52b	0.53b	0.54b
CV (%)	0.787	0.670	0.851	0.776	0.867
LSD_0.1%_	0.004	0.004	0.005	0.004	0.005
Level of concentrations					
50^0^B sucrose	0.51a	0.53a	0.55a	0.57a	0.60a
45^0^B sucrose + 5% NaCl	0.49b	0.51b	0.52b	0.54b	0.56b
5% NaCl	0.48c	0.50c	0.51c	0.53c	0.55c
CV (%)	0.787	0.670	0.851	0.776	0.867
LSD_0.1%_	0.005	0.004	0.006	0.005	0.006
Treatments					
T_1_	0.514a	0.534a	0.559a	0.594a	0.626a
T_2_	0.508ab	0.527b	0.547b	0.559b	0.578b
T_3_	0.511a	0.531ab	0.555ab	0.591a	0.619a
T_4_	0.503b	0.517c	0.531c	0.547c	0.571b
T_5_	0.479c	0.481d	0.493d	0.517d	0.545c
T_6_	0.4566	0.460e	0.467e	0.471e	0.479d
CV (%)	0.787	0.670	0.851	0.776	0.867
LSD_0.1%_	0.007	0.006	0.008	0.008	0.009

Note:

All values are means of triplicate determinations. Means within columns with different letters a, b, c, d, e indicates significant result (*p* ˂ .001).

Abbreviations: CV, Coefficient of variation; LSD, Least standard deviation; T_1_, 50^0^B sucrose in whole plum; T_2_, 50^0^B sucrose in peeled plum; T_3_, 45^0^B sucrose + 5% NaCl in whole plum; T_4_, 45^0^B sucrose + 5% NaCl in peeled plum; T_5_, 5% NaCl in whole plum; T_6_, 5% NaCl in peeled plum.

The color of osmo‐dehydrated plums is an important quality parameter. Color values of *L* (lightness), a* (redness), and b* (yellowness) of the initial and three‐month intervals up to twelve months stored plums are depicted in Table 4. The peeled plum obtained the highest lightness compared to the whole plum, and the trend of decreasing lightness continued even after 12 months of storage. Concerning the osmotic reagents and their concentration effect, it was observed that the highest lightness was found in the 50^0^B sucrose treated plums. For the interactive effects of peeling conditions and solute concentrations, the highest lightness was found in the treatment T_6_ and the second‐highest was in the treatment T_4_. The reduction of lightness during storage may be explained by the degradation of thermo‐labile pigments happening during the formation of dark compounds that blow up luminosity, and nonenzymatic browning reaction because of heat effect as reported by Dutta et al. (2006) and Goncalves et al. (2007). In the case of color coordinates a*, the highest values were found in the peeled plum and the lowest were observed in the whole plum considering the effect of peeling used as treatments. In the case of sucrose–NaCl concentrations, using 5% NaCl scored the highest values of color coordinate a*. For treatment interactions as the peeling conditions and the level of sucrose–sodium chloride concentrations, the highest values of a* were found in treatment T_4_ and the second‐highest was in treatment T_6_ and gradually it was decreased up to 12 months of storage. Initially, the plum color was red and it decreased slowly up to the end of the storage period concerning the color coordinates a*. For the color coordinates b*, it was observed that the highest values were found in the peeled plum and the lowest was in the whole plum due to the effect of sucrose–sodium chloride concentrations. With regard to the sucrose–sodium chloride concentrations, the 5% NaCl treated plums showed the highest values of b*. In the case of treatment interactions of peel conditions and solute concentrations, the highest color coordinates b* values were found in the treatment T_6_ followed by the treatment T_4_ and gradually it was decreased month by month during storage. The osmo‐dehydrated plum color was turned into yellowish to brownish color after 12 months of storage regarding color coordinates b*. This could be explained by the degradation of carotenoids in the plum tissue during storage (Miranda et al., 2009). The influence of temperature on heat‐sensitive compounds, such as carbohydrates, proteins, and vitamins, are responsible for the color degradation in fresh foods in addition to browning actions and pigment deterioration with drying processes (Hawlader et al., 2006; Maskan et al., 2002). Similar investigation has been pointed out by Prothon et al. (2001) for apples; Scala and Crapiste (2008) for red peppers; Koca et al. (2007) for carrots; and Vega et al. (2007) for red peppers. The plum color alterations might be explained by the carotenoid degradation by heat; nonenzymatic browning due to the degeneration of color. However, the effect of temperature on lightness and the coordinate was the same as that of on a* and b* values, meaning that the lightness of the osmo‐dehydrated plum was increased with the increasing of temperature (Adiletta et al., 2018).

**TABLE 4:** Effect of peeling and various solutes concentrations on the color parameters of osmo‐dehydrated plum during storage

Factors/Treatments	Color parameters of osmo‐dehydrated plum at different storage (months)				
	0	3	6	9	12
Lightness (L)					
Peeling conditions					
Whole plum	34.55b	31.81b	29.63b	26.46b	24.82b
Peeled plum	40.17a	36.46a	31.90a	28.87a	26.68a
CV (%)	0.888	0.905	0.933	0.959	0.946
LSD_0.1%_	0.348	0.324	0.302	0.279	0.256
Level of concentrations					
50^0^B sucrose	39.46a	33.15b	29.43b	27.08b	25.43c
45^0^B sucrose + 5% NaCl	37.26b	33.26b	29.79b	27.86a	25.76b
5% NaCl	35.36c	36.01a	33.09a	28.06a	26.08a
CV (%)	0.888	0.905	0.933	0.959	0.946
LSD_0.1%_	0.427	0.397	0.369	0.341	–
LSD_1.0%_	–	–	–	–	0.313
Treatments					
T_1_	31.13f	30.09e	28.74d	25.84d	24.01d
T_2_	37.84c	33.63c	29.93c	28.15b	26.19b
T_3_	35.85e	32.46d	30.51b	25.98d	24.49c
T_4_	39.59b	36.21b	30.12bc	28.32b	26.84a
T_5_	36.67d	32.89d	29.64c	27.57c	25.97b
T_6_	43.07a	39.55a	35.66a	30.13a	27.02a
CV (%)	0.888	0.903	0.933	0.959	0.946
LSD_0.1%_	0.603	0.561	0.522	0.483	0.443
Coordinates (a*)					
Peeling conditions					
Whole plum	14.26b	11.80b	10.70b	9.32b	7.75b
Peeled plum	22.73a	19.17a	16.33a	13.95a	12.06a
CV (%)	0.921	1.009	1.008	0.990	0.955
LSD_0.1%_	0.179	0.164	0.143	0.121	0.099
Level of concentrations					
50^0^B sucrose	17.88c	14.79b	12.80b	11.21b	9.68b
45^0^B sucrose + 5% NaCl	18.12b	14.42c	12.74b	10.55c	8.85c
5% NaCl	19.50a	17.24a	15.01a	13.17a	11.20a
CV (%)	0.921	1.009	1.008	0.990	0.955
LSD_0.1%_	0.219	0.201	0.175	0.148	0.122
Treatments					
T_1_	11.70f	9.46f	8.54f	7.26f	6.03f
T_2_	21.61c	17.92c	15.45c	12.11c	10.55c
T_3_	16.47d	15.01c	13.54d	11.73d	10.09d
T_4_	24.05a	20.12a	17.06a	15.145a	13.32a
T_5_	14.62e	10.92e	10.03e	8.98e	7.14e
T_6_	22.53b	19.46b	16.47b	14.60b	12.31b
CV (%)	0.936	1.001	1.020	0.980	0.958
LSD_0.1%_	0.315	0.282	0.251	0.207	0.173
Coordinates (b*)					
Peeling conditions					
Whole plum	13.81b	11.33b	9.41b	7.79b	6.84b
Peeled plum	20.77a	16.90a	14.52a	12.50a	11.08a
CV (%)	0.788	0.816	0.791	0.895	0.896
LSD_0.1%_	0.143	0.121	0.099	0.095	0.084
Level of concentrations					
50^0^B sucrose	18.02b	14.58b	12.85b	10.89a	9.28b
45^0^B sucrose + 5% NaCl	15.02c	12.47c	9.95c	8.96c	8.16c
5% NaCl	18.84a	15.29a	13.11a	10.57b	9.45a
CV (%)	0.788	0.816	0.791	0.895	0.896
LSD_0.1%_	0.175	0.148	0.122	0.117	0.103
Treatments					
T_1_	14.87d	12.10d	9.98d	8.40d	7.54d
T_2_	17.02c	14.22c	11.42c	10.53c	9.98c
T_3_	13.54e	11.17e	9.79e	7.56e	6.66e
T_4_	21.17b	17.06b	15.71b	13.39b	11.02b
T_5_	13.01e	10.73f	8.47f	7.40e	6.33f
T_6_	24.13a	19.42a	16.43a	13.58a	12.23a
CV (%)	2.521	0.808	0.793	0.882	0.865
LSD_0.1%_	0.793	0.207	0.173	0.163	0.141

Note:

All values are means of triplicate determinations. Means within columns with different letters a, b, c, d, e, & f indicates significant result (*p* ˂ .001 & ˂.01).

Abbreviations: CV, Coefficient of variation; LSD, Least standard deviation; T_1_, 50^0^B sucrose in whole plum; T_2_, 50^0^B sucrose in peeled plum; T_3_, 45^0^B sucrose + 5% NaCl in whole plum; T_4_, 45^0^B sucrose + 5% NaCl in peeled plum; T_5_, 5% NaCl in whole plum; T_6_, 5% NaCl in peeled plum.

**FIGURE 2:**
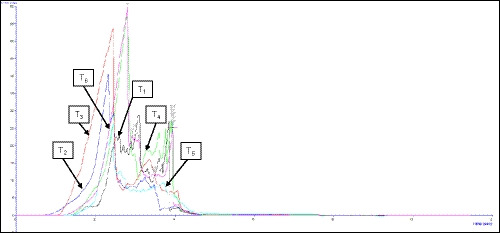
Texture of osmo‐dehydrated plum after 12 months of storage (Force vs Time). T_1_, 50^0^B sucrose in whole plum; T_2_, 50^0^B sucrose in peeled plum; T_3_, 45^0^B sucrose + 5% NaCl in whole plum; T_4_, 45^0^B sucrose + 5% NaCl in peeled plum; T_5_, 5% NaCl in whole plum; T_6_, 5% NaCl in peeled plum

The effect of peeling and solute concentrations on the texture of osmo‐dehydrated plum during storage are given in Table 5, and the texture profile of osmo‐dehydrated plum after 12 months of storage is shown in Figure 2. As shown in the Table, initially the texture of the peeled plums was 2.42 N‐mm^−2^ and that of the whole plum was 2.22 N‐mm^−2^. It was observed that the texture of the plum changed significantly due to different concentrations of sucrose–NaCl in the treatments. The highest texture of 2.51 N‐mm^−2^ was observed in only 5% NaCl plums and the lowest 2.08 N‐mm^−2^ was in the 50^0^B sucrose treated plums. However, the texture was gradually decreased after 12 months of storage. In connection with the interaction between peeling condition and concentrations, the highest texture of 2.77 N‐mm^−2^ was seen in treatment T_6_ and the lower of 1.79 N‐mm^‐2^ in treatment T_1_. The lower values of texture indicated that the good quality of osmo‐dehydrated plums. The texture reduction may be associated with the degradation of components responsible for the structural rigidity of the fruit, mainly insoluble pectin and protopectin discussed by Maftoonazad et al. (2008). The higher texture conservation in pretreated samples along the storage time can be attributed to the use of different sucrose–NaCl concentration in the osmotic dehydration as well as the peeling condition; the same result was investigated by Cristhiane et al. (2013) for fresh‐cut melon.

**TABLE 5:** Effect of peeling and various sucrose–sodium chloride concentrations on the texture (N‐mm^‐2^) of osmo‐dehydrated plum during storage

Factors/Treatments	The texture of osmo‐dehydrated plum at different storage (months)				
	0	3	6	9	12
Peeling conditions					
Whole plum	2.22b	1.70b	1.53b	1.44b	1.35b
Peeled plum	2.42a	1.86a	1.66a	1.55a	1.42a
CV (%)	1.467	1.331	2.018	1.862	2.586
LSD_0.1%_	0.036	0.025	0.034	0.029	‐
LSD_1.0%_	–	–	–	–	0.038
Level of concentrations					
50^0^B sucrose	2.08c	1.65c	1.50c	1.40c	1.34b
45^0^B sucrose + 5% NaCl	2.38b	1.77b	1.55b	1.51b	1.39a
5% NaCl	2.51a	1.92a	1.75a	1.59a	1.43a
CV (%)	1.467	1.331	2.018	1.862	2.586
LSD_0.1%_	0.044	0.030	0.041	0.036	–
LSD_1.0%_	–	–	–	–	0.046
Treatments					
T_1_	1.79f	1.49f	1.35e	1.29e	1.26e
T_2_	2.14e	1.61e	1.41d	1.41d	1.34d
T_3_	2.25d	1.69d	1.57c	1.44d	1.35 cd
T_4_	2.36c	1.81c	1.65b	1.51c	1.41bc
T_5_	2.62b	1.92b	1.68b	1.60b	1.44ab
T_6_	2.77a	2.15a	1.93a	1.73a	1.50a
CV (%)	1.467	1.331	2.018	1.862	2.586
LSD_0.1%_	0.062	0.043	0.059	0.051	0.065

Note:

All values are means of triplicate determinations. Means within columns with different letters a, b, c, d, e, and f indicates significant result (*p* ˂ .001 & ˂.01).

Abbreviations: CV, Coefficient of variation; LSD, Least standard deviation; T_1_, 50^0^B sucrose in whole plum; T_2_, 50^0^B sucrose in peeled plum; T_3_, 45^0^B sucrose + 5% NaCl in whole plum; T_4_, 45^0^B sucrose + 5% NaCl in peeled plum; T_5_, 5% NaCl in whole plum; T_6_, 5% NaCl in peeled plum.

The changes in sugar (reducing and total) of stored osmo‐dehydrated plum because of the effect of peeling and various sucrose–NaCl concentrations are depicted in Table 6. The fresh plum TSS was 8.9. Concerning the effect of peeling condition, it was observed that the highest content of reducing sugar of 26.42 was found in the whole plum and the lowest was 24.03 in the peeled plum. However, it was decreased month by month up to 12 months of storage. Coming to the effect of concentrations, the highest reducing sugar of 34.92 was observed in 50^0^B sucrose followed by 22.12 which was found in 45^0^B sucrose + 5% NaCl concentration. As for the interaction between peeling condition and concentrations, initially, the highest reducing sugar of 41.66 was seen in treatment T_1_ and the lowest value was 39.13 in treatment T_2_. Interestingly, reducing sugar was gradually decreased after 12 months of storage. For the total sugar content of the osmo‐dehydrated plums, the highest content in the whole plum was 43.12 and the lowest was 40.35 in the peeled plum as a part of the peeling effect. Concerning the effect of solute concentrations, plum dipped into 50^0^B sucrose showed the highest content of sugar of 59.68 which was followed by the values of 35.13 for 45^0^B sucrose + 5% NaCl concentration. Concerning the interaction between peeling condition and sucrose–NaCl concentrations variation, the total sugar content for only sucrose treated plum was initially 68.12 and 64.51 for the T_1_ and T_2_ treatments, respectively, but after 12 months of storage it was decreased to 41.66 and 39.70, respectively. The total sugar was decreased by 38.84% and 38.46 percent for the treatments of T_1_ and T_2_, respectively. Nevertheless, in the beginning, the total sugar content of the NaCl treated osmo‐dehydrated plum was 5.74 and 5.32 in the treatments of T_5_ and T_6_, respectively; subsequently, after 12 months of storage, it was decreased to 5.19 and 4.72, respectively. The reduction of total sugar content of NaCl treated plum was 9.58% and 11.28 percent for the treatments T_5_ and T_6_, respectively. The observed variation was due to increase in moisture content and might also be due to conversion of sugar due to nonenzymatic browning reactions in the osmo‐dehydrated plum (Nazaneen et al., 2015; Tomar et al., 1990). Sugar content in various treated plums varied significantly due to the variation of the sucrose–NaCl concentrations during osmotic treatments and peel conditions. As sucrose is used in plum, an increase in the content of sucrose makes the plum more caloric. For the reduction of the energy value of dried plums, sodium chloride can be used as an osmotic agent and a similar result was found by Robert (2008). The plums treated with a higher percentage of sucrose along with peeling attributed to the higher values of reducing sugar and total sugar. This might be due to the effect of sugar syrups used for osmosis and the expose of the flesh of the plum after removal of the peel (Kumar & Sagar, 2014). The osmo‐dehydrated plum gives a higher percentage of sucrose when sucrose is used as an osmotic agent as reported for the dehydrated mango slices and osmo‐dried apple rings, respectively, during storage (Kumar, 2013).

**TABLE 6:** Effect of peeling and various sucrose–sodium chloride concentrations on the reducing sugar and total sugar of osmo‐dehydrated plum during storage

Factors/Treatments	The sugar content of osmo‐dehydrated plum at different storage (months)								
	0	3		6		9		12	
Reducing sugar (%)									
Peeling conditions									
Whole plum	26.42a		23.33a		20.62a		19.63a		18.74a
Peeled plum	24.03b		21.07b		19.04b		18.95b		18.05b
CV (%)	1.197		1.252		1.033		0.889		0.680
LSD_0.1%_	0.317		0.292		0.215		0.180		0.131
Level of concentrations									
50^0^B sucrose	34.92a		30.58a		27.69a		26.48a		25.66a
45^0^B sucrose + 5% NaCl	22.12b		18.72b		16.59b		16.77b		15.18b
5% NaCl	18.65c		17.30c		15.22c		14.64c		14.35c
CV (%)	1.197		1.252		1.033		0.889		0.680
LSD_0.1%_	0.389		0.357		0.264		0.221		0.161
Treatments									
T_1_	41.66a		35.13a		31.25a		29.41a		27.30a
T_2_	39.13b		32.72b		28.72b		29.17a		26.11b
T_3_	32.5c		30.14c		26.15c		25.12b		24.67c
T_4_	28.17d		26.03d		24.13d		23.54c		24.01d
T_5_	5.10e		4.72e		4.46e		4.37d		4.25e
T_6_	4.79e		4.45e		4.28e		4.15d		4.02e
CV (%)	1.189		1.274		1.008		0.898		0.769
LSD_0.1%_	0.546		0.515		0.364		0.315		0.257
Total sugar (%)									
Peeling conditions									
Whole plum	43.12a		39.22a		34.73a		30.61a		27.95a
Peeled plum	40.35b		36.71b		33.31b		28.66b		27.06b
CV (%)	0.915		0.882		0.648		1.089		0.888
LSD_0.1%_	0.401		0.352		0.232		0.339		0.257
Level of concentrations									
50^0^B sucrose	59.68a		54.01a		49.10a		43.05a		39.21a
45^0^B sucrose + 5% NaCl	35.13b		30.85b		27.18b		23.43b		22.45b
5% NaCl	30.41c		29.04c		25.79c		22.40c		20.87c
CV (%)	0.915		0.882		0.648		1.089		0.888
LSD_0.1%_	0.491		0.431		0.284		0.415		0.314
Treatments									
T_1_	68.12a		59.13a		52.14a		46.54a		41.66a
T_2_	64.51b		56.23b		49.01b		41.56b		39.7b
T_3_	55.50c		53.06c		46.71c		40.01c		37.01c
T_4_	51.23d		48.89d		46.06d		39.56c		36.76c
T_5_	5.74e		5.47e		5.35e		5.29d		5.19d
T_6_	5.32e		5.01e		4.87f		4.79d		4.72e
CV (%)	0.758		0.833		0.657		1.067		0.891
LSD_0.1%_	0.575		0.575		0.407		0.575		0.446

Note:

All values are means of triplicate determinations. Means within columns with different letters a, b, c, d, e, and f indicates significant result (*p* ˂ .001).

Abbreviations: CV, Coefficient of variation; LSD, Least standard deviation; T_1_, 50^0^B sucrose in whole plum; T_2_, 50^0^B sucrose in peeled plum; T_3_, 45^0^B sucrose + 5% NaCl in whole plum; T_4_, 45^0^B sucrose + 5% NaCl in peeled plum; T_5_, 5% NaCl in whole plum; T_6_, 5% NaCl in peeled plum.

The changes in total phenolic contents of stored osmo‐dehydrated plum are presented in Table 7. For the effect of peeling, it was observed that the total phenolic content of 889.78 mg/100 g was found as the highest in the peeled plum and 860.78 mg/100 g as the lowest in the whole plum during storage, and it was decreased slowly month by month. As to the effect of sucrose–sodium chloride concentrations, at beginning the highest total phenol of 937.61 mg/100 g was observed using only 5% NaCl concentrations which was followed by the value of 859.60 for the 45^0^B sucrose + 5% NaCl concentration. The interaction between peeling condition and various solute concentrations, initially, the highest total phenol of 990.05 mg/100 g was seen in treatment T_6_ and the lowest of 723.06 mg/100 g in treatment T_1_. Finally, the total phenolic content was slightly decreased after 12 months of storage at room temperature. It was happened because of the slower enzymatic reactions in dried plum at a lower temperature of storage as the temperature is a major factor in the initiation and feasibility of a chemical reaction. The phenolic contents occur to produce yellowish to brownish color (Clifford, 2000; Kumar, 2013) at different transformations for the time of food processing. Generally, the dried plum showed higher total phenolic contents as compared to the fresh plum (356 mg/100 g) and the similar investigation observed by Stacewicz‐Sapuntzakis et al. (2001) and Dowling (2014).

**TABLE 7:** Effect of peeling and various solutes concentrations on the total phenol (mg/100g) of osmo‐dehydrated plum during storage

Factors/Treatments	Total phenol of osmo‐dehydrated plum at different storage (months)				
	0	3	6	9	12
Peeling conditions					
Whole plum	860.78b	765.09b	686.43b	623.25b	566.74b
Peeled plum	889.80a	797.31a	714.82a	645.79a	585.43a
CV (%)	0.528	0.528	0.500	0.475	0.420
LSD_0.1%_	4.859	4.329	3.681	3.169	2.541
Level of concentrations					
50^0^B sucrose	828.67c	744.14c	654.67c	583.25c	513.83c
45^0^B sucrose + 5% NaCl	859.60b	765.42b	703.14b	643.51b	598.88b
5% NaCl	937.61a	834.04a	744.06a	676.81a	615.55a
CV (%)	0.528	0.528	0.500	0.475	0.420
LSD_0.1%_	5.951	5.302	4.509	3.881	3.112
Treatments					
T_1_	723.06f	647.12f	598.03e	537.26e	484.50f
T_2_	745.07e	663.71e	602.14e	542.23e	492.32e
T_3_	885.17d	781.01d	657.11d	587.70d	510.28d
T_4_	934.27c	841.16c	711.31c	629.24c	543.16c
T_5_	974.12b	867.13b	804.14b	744.79b	705.44b
T_6_	990.05a	887.07a	831.01a	765.91a	720.81a
CV (%)	0.524	0.528	0.494	0.473	0.425
LSD_0.1%_	8.337	7.501	6.302	5.458	4.456

Note:

All values are means of triplicate determinations. Means within columns with different letters a, b, c, d, e, and f indicates significant result (*p* ˂ .001).

Abbreviations: CV, Coefficient of variation; LSD, Least standard deviation; T_1_, 50^0^B sucrose in whole plum; T_2_, 50^0^B sucrose in peeled plum; T_3_, 45^0^B sucrose + 5% NaCl in whole plum; T_4_, 45^0^B sucrose + 5% NaCl in peeled plum; T_5_, 5% NaCl in whole plum; T_6_, 5% NaCl in peeled plum.

The dehydrated plum overall acceptability by the consumer is highly dependent on its sensory attributes. In addition to visual appearance, color, flavor, and textural attributes are critical in determining their degree of acceptance. The organoleptic attributes of the osmo‐dehydrated plum with different combinations of sucrose–sodium chloride concentrations as well as the conditions of peeling were assessed after three months interval up to twelve months of storage. Comparative sensory evaluation of different quality attributes of the osmo‐dehydrated plums according to the opinion of taste panel judges comprising 10 members are presented in Table 8. It was observed that the color, flavor, taste, sweet–sour balance, and bitterness had a significant effect on its overall acceptance. According to the Table, it was observed that the overall acceptability of 7.17 was found as the highest for the whole plum and 6.67as the lowest for the peeled plum. As for the effect of concentrations, initially, the highest overall acceptability of 7.75 was observed in 50^0^B sucrose and followed by the value of 6.75 for 45^0^B sucrose + 5% NaCl treated plum. With regard to the interaction between peeling conditions and concentrations, initially, the highest overall acceptability of 8.50 was investigated in treatment T_1_ and 8.0 was in treatment T_2_ securing the second‐highest score. Finally, the highest overall acceptability was continued in treatment T_1_ up to the end of storage and it was 8.0 (i.e., like very much) that was judged by the panelists. Panelists liked the osmo‐dehydrated plums because of the balance of sodium chloride‐sucrose percentage, less bitterness, attractive color, and overall taste as mentioned during judgment. The best color of the osmo‐dried plum might be owing to the effect of KMS used in different treatments as well as the color retained due to the faster dehydration of the treated plum (Ahrne et al., 2003; Akpinar & Bicer, 2005).

**TABLE 8:** Effect of peeling and various solutes concentrations on the overall acceptability of osmo‐dehydrated plum during storage

Factors/Treatments	Overall acceptability of osmo‐dehydrated plum at different storage (months)				
	0	3	6	9	12
Peeling conditions					
Whole plum	7.17a	7.08a	6.92a	6.67a	6.50a
Peeled plum	6.67b	6.58b	6.17b	6.17b	5.92b
CV (%)	5.593	4.984	4.603	3.688	2.774
LSD_0.1%_	–	–	0.316	–	0.181
LSD_1.0%_	–	–	–	0.249	–
LSD_5.0%_	0.406	0.358	–	–	–
Level of concentrations					
50^0^B sucrose	7.75a	7.63a	7.50a	7.25a	7.13a
45^0^B sucrose + 5% NaCl	6.75b	6.63b	6.25b	6.13b	5.88b
5% NaCl	6.25c	6.25b	5.88b	5.88b	5.63c
CV (%)	5.593	4.984	4.603	3.688	2.774
LSD_0.1%_	0.498	0.438	0.387	0.304	0.222
Treatments					
T_1_	8.50a	8.50a	8.50a	8.00a	8.00a
T_2_	8.00ab	8.00ab	7.50b	7.50b	7.25b
T_3_	7.50bc	7.50b	7.25b	7.25b	7.00b
T_4_	7.00c	6.75c	6.50c	6.50c	6.25c
T_5_	5.50d	5.25d	5.00d	4.75d	4.50d
T_6_	5.00d	5.00d	4.50d	4.50d	4.25d
CV (%)	5.594	4.984	4.604	3.688	2.776
LSD_0.1%_	0.704	0.620	0.548	0.431	0.314

Note:

All values are means of triplicate determinations. Means within columns with different letters a, b, c, and d indicates significant result (*p* ˂ .001, ˂.01 & ˂.05).

Abbreviations: CV, Coefficient of variation; LSD, Least standard deviation; T_1_, 50^0^B sucrose in whole plum; T_2_, 50^0^B sucrose in peeled plum; T_3_, 45^0^B sucrose + 5% NaCl in whole plum; T_4_, 45^0^B sucrose + 5% NaCl in peeled plum; T_5_, 5% NaCl in whole plum; T_6_, 5% NaCl in peeled plum.

## 4 CONCLUSIONS

The research results were analyzed under the parameters of drying kinetics, rehydration properties, water activity, color, texture, sugar, total phenol, and overall acceptability of the osmo‐dehydrated plum through sensory evaluation to assess the drying kinetics and the quality attributes of the osmo‐dehydrated plum prepared from fresh plum during one‐year storage in an ambient condition. The osmo‐dehydrated plum prepared from whole plums osmosed in 50^0^B sucrose solution performed better considering the dehydration kinetics and analysis of the different quality attributes of the plums even after 12 months of storage at room temperature. Therefore, the developed technique would be helpful for the farmers/growers and traders for preparing osmo‐dehydrated plum from fresh plum to prevent postharvest losses in addition to fulfill nation demand.

## CONFLICT OF INTEREST

The author(s) declared no conflicts of interest with the research, authorship, and publication of this article.

## ETHICAL APPROVAL

This research does not involve any human or animal testing.

## Data Availability

The data that support the findings of this study are available from the corresponding author upon reasonable request.
